# Promising Strategies in Plant-Derived Treatments of Psoriasis-Update of In Vitro, In Vivo, and Clinical Trials Studies

**DOI:** 10.3390/molecules27030591

**Published:** 2022-01-18

**Authors:** Martyna Nowak-Perlak, Krzysztof Szpadel, Izabella Jabłońska, Monika Pizon, Marta Woźniak

**Affiliations:** 1 Department of Clinical and Experimental Pathology, Division of General and Experimental Pathology, Wroclaw Medical University, 50-368 Wroclaw, Poland; martyna.nowak-perlak@student.umw.edu.pl (M.N.-P.); krzysztof.szpadel@student.umed.wroc.pl (K.S.); izabella.jablonska@student.umed.wroc.pl (I.J.); 2 Transfusion Center Bayreuth, 95448 Bayreuth, Germany; mpizon@simfo.de

**Keywords:** natural substances, medicinal plants, psoriasis, antioxidants

## Abstract

Psoriasis is a common, chronic systemic inflammatory disease affecting 125 million people worldwide. It is associated with several important conditions, including psoriatic arthritis, cardiometabolic syndrome, and depression, leading to a significant reduction in patients’ quality of life. Current treatments only reduce symptoms, not cure. This review discusses the mechanisms involved in the initiation and development of the disease, the role of oxidative stress in this autoimmune disease, as well as potential therapeutic options with substances of natural origin. The main aim of the study is intended to offer a review of the literature to present plants and phytochemicals that can represent potential remedies in the fight against psoriasis. We identified many in vitro, in vivo, and clinical trials studies that evaluated the relationship between chosen natural substances and immune system response in the course of psoriasis. We sought to find articles about the efficacy of potential natural-derived drugs in controlling symptoms and their ability to maintain long-term disease inactivity without side effects, and the result of our work is a review, which highlights the effectiveness of plant-derived drugs in controlling the inflammatory burden on psoriatic patients by decreasing the oxidative stress conditions.

## 1. Introduction

Psoriasis is a chronic, autoimmunologic disease known since ancient times [1]; as the most common genetic skin disorder, it is mainly manifested by gradually magnifying and exfoliating psoriatic plates with accompanying pustules and spots [2]. The first lesions occur in the highest layer of the dermis–the papillary dermis. Blood vessels become dilated and curvy, and neutron-absorbing lymphocytes and granulocytes emerge from the light and reach the epidermis, which at this stage still looks quite ordinary. Herein the abnormal proliferation and migration of keratinocytes begin. Subsequently, the epidermis thickens, and keratinocytes stop to differentiate completely, which leads to loss of a granular layer and occurrence of parakeratosis (keratosis of the stratum corneum, induced by nuclei bearing keratinocytes). With psoriasis progression, some skin cell populations excessively proliferate, expanding the spinous layer of epithelium and leading, i.e., to acanthosis nigricans. The epidermis’s stratum corneum completely disappears, the granular layer disappears, T cells with glycoprotein 8 are dispersed between keratinocytes, and neutron-absorbent granulocytes accumulate in parakeratotic plates, forming Munro’s microabscesses. Dilated blood vessels extend to the highest layer of the dermis, causing bleeding after removing psoriatic plaque [3]. Usually, this type of psoriasis appears as red patches of inflamed skin, raised, coated with silvery-white scales. Often, the patches show in a symmetrical pattern.

Psoriasis is classified as an autoimmune disease caused by malfunctioning pathways and elements of the immune system T cells, dendritic cells, cytokines such as interleukin-23, interleukin-17, and tumor necrosis factor [4]. It is the most common genetic skin disease, which leads to the increased risk of developing metabolic syndrome (MS) and cardiovascular disease. Moreover, severe psoriasis patients present increased chances for the development of components of metabolic syndrome as obesity, dyslipidemia, diabetes mellitus, and hypertension [5,6,7].

Presently, most of the conventional therapies can diminish psoriasis symptoms. However, there is not yet a known treatment that could cure this condition completely. Furthermore, many of those strategies can cause various side effects among patients, such as atrophy, organ toxicity, immunosuppression, infection, and carcinogenesis, limiting these therapies’ application in long-term use. Hence, further development of safe, effective, and possibly less expensive methods of treating psoriasis is needed.

Research reveals that one of the possible ways to modulate the response of the cells engaging in the psoriasis course is to use herbal drugs and exploit their immunoregulatory and antioxidative role in the treatment. Literature reviews document the usefulness of herbal remedies for psoriasis and the supportive role of phytochemicals in this autoimmune disease treatment [8,9], but the novelty of this studies presents a detailed and updated discussion of the antioxidative properties of plant-derived substances throughout the different stages of the studies: in vitro, in vivo, and in clinical trials. Moreover, this review shows the natural plant compounds and their various effect on oxidative stress pathways.

This detailed review discusses the scientific evidence regarding the influence of plant-derived substances (phytochemicals and plant based extracts) on immune system compounds responsible for the inflammation and the course of psoriasis, as well as the potent role of natural substances in the reduction of oxidative stress, which triggers the activation of several signaling pathways responsible for the development of this autoimmune disease.

Moreover, the review presents the different in vitro, in vivo, and clinical studies with plant-derived compounds and investigates their role in the treatment of psoriatic symptoms and their immunomodulatory and anti-inflammatory effect on the immune cell populations and cytokines responsible for psoriasis development. To better understand the role of natural derived substances and present their potency in psoriasis treatment, this article comprises detailed data about the pathogenesis of this autoimmune disease in regards to inflammation process and oxidative stress, as well as the molecular interactions between natural active compounds and immune system response.

## 2. Epidemiology and Pathophysiology of Psoriasis

The average incidence of psoriasis worldwide is around 2%. The illness is more common in Caucasian people, less in Asians, and least in Black people [10]. Psoriasis is more common in populations living in cold northern regions and less often in communities living in tropical climates. In Europe, psoriasis affects 0.6–6.5% of people and is more common in north countries [11].

Regardless of age, psoriasis can occur in any person. It is difficult to ascertain the average age at which the disease most frequently develops due to somewhat late clinic visits and the fact that the disease’s first symptoms may have occurred even a few years before the first medical appointment. Several studies have shown that the disease’s first signs will appear between the ages of 15 and 20 at the earliest and between 55 and 60 at the latest [12,13].

Because of the complexity of this disease that involves skin, nails, joints, adaptive and innate immune system responses, and may appear in different psoriasis subtypes according to the pathomechanisms, psoriasis can be characterized by extensive pathophysiology concepts. Nowadays, it is well-known that autoimmune-related pathomechanisms connected to immune system cells and cytokines are the main target for psoriasis treatment.

Another notion about the psoriasis pathophysiology is connected with skin microbiota. Different bacteria that colonize patients’ skin are involved in immune system regulation. Therefore, it is hypothesized that an aberrant immune activation triggered by skin microbiota plays a role in autoimmune diseases pathogenesis [14].

In addition, genetic predispositions to inherit psoriasis are also important factors, and the profiling of gene expression in psoriasis may help in understanding the pathophysiology of this disease. Genetic mutations in immune cell regulatory proteins affect different cytokine expression, which in turn influence the proliferation and differentiation of keratinocytes. To conclude, the genetic variants of immune cells and proteins associated with psoriasis are responsible for different courses of psoriasis [15].

One of the last concepts of psoriasis pathophysiology has the root in the epigenetic modifications: DNA methylation, histone modifications, and non-coding RNAs (via microRNAs and long non-coding RNAs). Some research presents a detailed discussion about the changes of gene expression as a consequence of the chemical modifications of DNA and histones, which alter chromatin structure, and thus, transcription factors of different genes [16,17].

## 3. Immune System Response in Psoriasis

The etiology of psoriasis is very complicated, mainly due to the skin’s abnormal immune response, resulting from genetic factors and various environmental stimuli, for example, skin injuries, infections, and drugs taken [18].

A characteristic feature of psoriasis is long-term inflammation that leads to the uncontrolled proliferation of keratinocytes and their abnormal differentiation. The histology of psoriatic plaque shows the hyperplasia of the epidermis, which results in inflammation composed of dermal dendritic cells, macrophages, T cells, and neutrophils [19].

**Figure 1:**
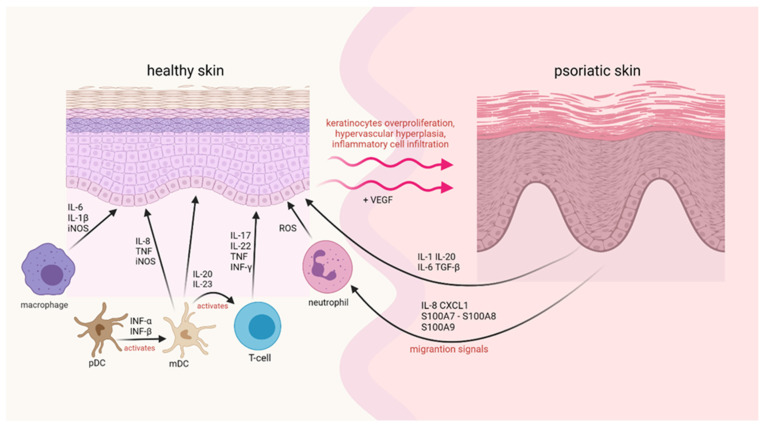
The scheme presents complicated mechanisms of the psoriasis course. Proinflammatory cytokines and other factors overproduced in psoriasis contribute to the increased proliferation of keratinocytes. Moreover, in psoriasis conditions, activation of inflammatory cells occurs and stimulates intense inflammation. These self-perpetuating loops of proinflammatory activators and effectors enhance the symptoms of this autoimmune disease.

In psoriatic plaque formation, two different types of cells can be found—keratinocytes derived from the epidermis and mononuclear leukocytes. The regulation of gene expression in these cell types is influenced by the other genes responsible for psoriasis [7]. Keratinocytes act on the immune system and activate leukocytes in psoriatic changes. There are two sets of cellular responses in psoriatic changes, which disturb the balance between the activation of innate and acquired types of immune cells and the factors produced by keratinocytes, directly affecting T cells and dendritic cells (Figure 1) [1].

Effector cells of congenital immunity in psoriatic changes include neutrophils, plasmacytoid dendritic cells (pDC), and cd11c+ dendritic cells. Since neutrophils live quite briefly, they must be continuously produced in the bone marrow and transported to the blood. Chemokines such as interleukin-8 (IL-8), CXCL1, and proteins like S100A7/A8/A9 derived from keratinocytes produce a chemotactic gradient for neutrophil migration to the epidermis [1]. PDCs with BDCA-2+ and CD123+ antigen expression, which makes high levels of interferon-α (IFN-α) after activation, are believed to play a crucial role in triggering disease changes [20]. The level of CD11c+ dendritic cells is increased in psoriatic changes [21].

Langerhans cells have long been recognized as the main type of DC in the skin [22]. Nevertheless, it is already known that in psoriatic changes, we observe additional types of DC. CD11c+ myeloid dendritic cells correspond to interstitial DC in other tissues and are the most common DC type in the dermis, whose levels additionally increase in psoriatic changes [21]. In psoriatic lesions, DC CD11c+ is characterized by high levels of tumor necrosis factor (TNF) and nitric oxide synthesis (iNOS) [23]. Besides, DC CD11c+ is likely to produce proinflammatory cytokines such as IL-23 and IL-20, potentially activating T cells and keratinocytes [24]. Part of DC CD11c+ has “maturation” markers, such as DC-LAMP or CD83. They can function as DC to present antigens to T cells to trigger acquired immune responses [25].

The abundance of T cells and mature DC in skin aggregates, combined with the expression of lymphoid-forming chemokines such as CCL19, CCL21, CXCL12, and CCL18 [26], may promote the activation of T cells in situ [27]. T cells in psoriatic changes are divided into two groups due to their functions-auxiliary T cells (T_H_) and cytotoxic T cells (T_C_) [28]. Some T cells express CD161 and other killer receptors, indicating the role of natural deadly T cells in psoriasis pathogenesis [29]. The products of keratinocytes perpetuate immune activation, and activated immunocyte products alter keratinocyte responses, including induction of new adhesive molecules for T cells. Heat shock proteins (HSP) or S100A12 produced by Toll-like receptors or agonists (TLR) can initiate DC activation and maturation. Peptide antigens may also trigger conventional or acquired immune activation of T cells, suggesting the presence of clonal T-cell populations in psoriatic changes [30].

Chronic immune activation and the faulty regulatory role of T cells can be explained by antigen. Their presence is another factor that can lead to rampant T cells activation [31]. Perspectives to develop effective therapeutic measures through rational design depend primarily on explaining the molecular pathways of inflammation in human autoimmune diseases. Cytokine interactions in psoriasis can rely on the assumption that a linear relationship between inducers (IL-23 or IL-12), the production of IFN-γ and TNF by T cells, and the activation of multiple IFN-responsive genes via signal relay and transcription activator (STAT1) exists [26]. This model is conceptually useful, representing only a small part of the more than 1300 genes that are positively regulated in psoriatic changes.

TNF-α, lymphotoxin-α (LT-α), IL-1, IL-17, IL-20, IL-22, and IFN are the main cytokines that can trigger STAT or NF-κB transcription factors (involved in strengthening inflammation) [24]. Activated DC can work with IFN-α, IL-20, IL-12, and IL-23 in an inflammatory mechanism. Activation of T cells by IL-12 or IL-23 (blue arrow) leads to the synthesis of inflammatory cytokines derived from T cells. Cytokines, or heat shock proteins (HSP), and direct interaction with anti-receptors on T cells.

Other cytokines synthesized by keratinocytes or stromal cells are likely to control the growth and fibroplasia of epithelial-stromal (vascular) in psoriatic lesions. TGF-β, IL-1, IL-6, and IL-20 may act as autocrine and/or paracrine keratinocyte growth factors [32].

There is also a complex set of chemokine interactions because at least a dozen of them have increased expression in psoriatic changes, suggesting that there are many other pathways for regulating psoriasis [33].

## 4. Oxidative Stress in the Course of Psoriasis

During the last few years, numerous studies have shown evidence that both ROS and NOS are involved in the pathogenesis of psoriasis. Redox imbalance, as well as increased levels of inducible NOS, are responsible for the generation of oxidative stress. Thus, it is important to understand the molecular mechanisms of pathogenic inflammation caused by the above-mentioned molecules. This may shed light on targeting the oxidative stress dysregulated pathways with natural substances possessing antioxidative activity, which can be candidates for further study to obtain the therapy for this complex disease.

### 4.1. ROS

Reactive oxygen species are a collective group that includes any molecule that contains oxygen and can react with other substances [34]. It can be divided into radicals that include: hydrogen superoxide (HO_2_•), superoxide (O_2_•^−^), hydroxyl (OH•) and peroxyl radicals (RO_2_•); and non-radicals such as ozone (O_3_), hydrogen peroxide (H_2_O_2_) and hypochlorous acid (HOCl) that can easily be transformed into radicals [35].

Two electrons with opposite spins must react to form a bond. Molecular (triplet state) oxygen has two electrons on the last shell with the same spin, which makes it able to bind to only one electron. By inducing oxygen and reversing the spin of one electron, the newly formed ROS can react efficiently with other compounds, including those having a double bond [35,36,37].

ROS are produced in the cell by enzyme-controlled metabolism as well as by radiation and xenobiotics. The most common is the formation of ROS as a byproduct during intracellular respiration. During the electron transport chain, oxygen is gradually reduced to eventually combine with hydrogen to form a water molecule. ROS are also produced during this process and can be released from the mitochondria. Another source of ROS is metabolism in peroxisomes, which catabolizes biomolecules by removing hydrogen from them in an oxidation reaction, resulting in the formation of hydrogen peroxide. A very important enzyme is leukocyte NADPH oxidase, whose main function is the rapid production of ROS during the “oxygen burst” that is of immune significance. In the non-enzymatic process of ROS formation, part of the blame is placed on ultraviolet radiation that acts on the skin [35,36,38,39].

### 4.2. NOS

Nitric oxide synthases (NOS) are a group of enzymes that catalyze the synthesis of NO. In the human body, we can recognize three isoforms of NOS: eNOS–endothelial NOS, nNOS–neuronal NOS, and iNOS–inducible NOS. It is also possible to divide NOS based on the type of expression and the dependence of their activity on calcium ions. eNOS and nNOS are constitutive, that is, they are constantly present in the cell and show the connection to calcium ions, whereas iNOS synthesis is inducible and this isoform is not Ca^2+^ dependent [40,41].

In psoriasis, iNOS is overly synthesized by various cell types, including keratinocytes, compared to healthy skin cells. Because of the generation of oxidative stress by this isoform, iNOS is indicated as one of the factors in the pathogenesis of this disease [42]. In its active form, NOS is a homodimer whose substrates are NADPH, O_2_, and l-arginine, while cofactors include flavin mononucleotide (FMN), calmodulin, tetrahydrobiopterin (BH4), and flavin adenine dinucleotide (FAD). Initially, molecular oxygen and NADPH are used to hydroxylate l-arginine to Nω-hydroxy-l-arginine. In the second step, NOS catalyzes the oxidation of Nω-hydroxy-l-arginine to l-citrulline and NO [43,44].

At physiological concentrations, NO has regulatory functions, but when its concentration is increased and oxygen is present, reactive nitrogen species (RNS) are formed. Nitric oxide can react with superoxide resulting in the formation of ONOO^−^, a highly reactive oxidant that causes numerous metabolic changes [42,43].

Oxidative stress occurs when the ratio between reactive oxygen species (ROS) and antioxidants favors ROS, which causes redox signaling and regulation to be impaired, as well as several molecular abnormalities [45,46]. Studies show that oxidative stress induced by different levels of ROS causes DNA modification, lipid peroxidation, and the secretion of proinflammatory cytokines. ROS, being second messengers, influence cellular signaling pathways, chiefly proinflammatory signaling pathways, and the expression of a variety of genes [47,48]. Cellular damage is caused primarily by high levels of ROS, while their low levels play an important role in physiological cellular processes [49].

Increased production of ROS, reactive nitrogen species (RNS), and decreased concentration of antioxidants are some of the most important causes of the pathogenesis of several diseases, including psoriasis [50,51,52]. The human body can neutralize oxidative stress factors even before they make a negative impact on cells. Enzymes found in the skin, such as superoxide dismutase (SOD), catalase (CAT), and glutathione peroxidase (GPx), which are responsible for lowering ROS levels, play a key role in the antioxidant defense process [53,54]. The importance of non-enzymatic antioxidants, which include vitamin E, C, and glutathione (GSH), also should be emphasized [55]. Levels of SOD, CAT, and GPx in the course of psoriasis may vary in different cells and tissues, but levels of vitamin E (α-tocopherol) and GSH show a downward trend [56,57]. While severe oxidative stress causes cell death, recent research has shown that mild oxidative stress is more important than severe oxidative stress in the pathogenesis of psoriasis [51]. According to studies, there is a positive correlation between oxidative stress markers and the psoriasis area and severity index (PASI), and a negative one between antioxidant markers and PASI. These studies indicate that factors such as total oxidative stress (TOS), plasma or serum malondialdehyde (MDA) could serve as useful biomarkers for psoriasis [54,55,58,59,60].

In psoriasis, oxidative stress triggers the activation of several signaling pathways induced by TNFα, mainly nuclear factor kappa-light-chain-enhancer of activated B cells (NF-κB), the mitogen-activated protein kinases (MAPK), and Janus kinase signal transducer and activator of transcription JAK-STAT pathways. This results in the activation of Th cells (mostly Th1 and Th17), the release of inflammatory cytokines, cell proliferation, differentiation but also death in keratinocytes and fibroblasts, immune cell invasion into the skin, and changes in angiogenesis via lipid peroxidation [47,50,61,62,63].

The process of lipid peroxidation results in the increased level of cGMP and lower cAMP activity which causes excessive epidermal proliferation. Patients with psoriasis also have a considerable concentration of an oxidized fraction LDL (ox-LDL) and high activity of phospholipase A2 (PLA2) [64,65]. Furthermore, ROS increase cytosolic Ca^2+^ levels by interacting with them, resulting in the disruption of differentiation and proliferation processes, and may also lead to cell death [66].

The increased number of myeloid dendritic cells (mDCs) in psoriasis may drive the immunopathological process by releasing IL-8 and TNFα and promoting T-cell proliferation in response to oxidative stress [67,68,69]. Oxidative conditions also support Th1 cells development. Consequently, higher levels of Th1-type cytokine IFN-c and IL-2 are expressed in psoriatic skin lesions [70,71]. Moreover, ROS induces vascular endothelial growth factor (VEGF) releasing from many cell types, thus being a crucial factor in the angiogenesis in psoriasis [72,73]. Leukocyte migration through psoriatic skin also may be driven by this factor and enhance the inflammatory process [74,75].

ROS also activate subgroups of MAPKs: the extracellular signal-regulated kinases (ERKs), the c-Jun N-terminal kinases (JNKs), and the p38 MAPKs [76]. Data shows that these serine-threonine protein kinases play a major role in the development of psoriasis [77,78,79]. ROS can both activate and inhibit NF-κB signaling, depending on the situation. It has been proven that both active, phosphorylated NF-κB and ROS are significantly elevated in psoriasis which makes ROS an activating factor for NF-κB and a contributor to the pathogenesis of this inflammatory disease [80]. ROS also can activate JAK/STAT pathway in human lymphocytes what indicates that oxidative stress can play a role in psoriasis development by activating the JAK/STAT pathway [81].

## 5. Substances of Natural Origin—Plants and Phytochemicals with Potential Therapeutic Significance in Reduction of ROS and iNOS

**Figure 2:**
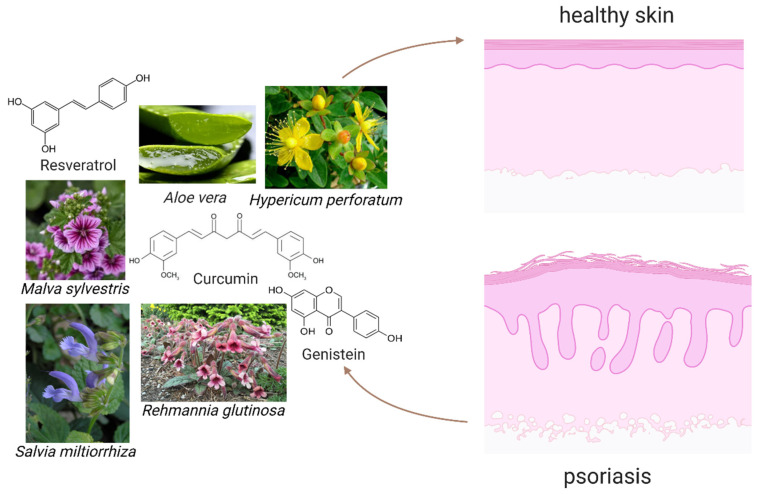
The scheme demonstrating the chosen plants and phytochemicals that may help to reduce symptoms of psoriasis, and act as antiproliferative compounds for psoriatic keratinocytes.

**Figure 3:**
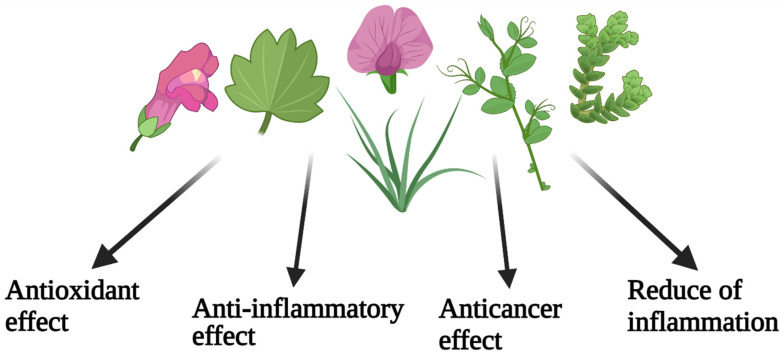
Schematic figure presenting the possible mechanisms of plant-derived compounds in order to treat psoriasis disease.

Recently, during the pursuit of novel therapies, natural compounds have gained significant attention due to their vast diversity, safety, and availability [82]. Several clinical studies have shown that some of the following natural origin substances (Figure 2) can attenuate psoriasis through numerous molecular mechanisms associated with apoptosis, inhibition of angiogenesis, and suppression of inflammation that is caused by reactive oxygen species and overexpression of inducible-nitric oxide synthases (Figure 3) [83,84].

### 5.1. Vegetal Medicinal Species

#### 5.1.1. Aloe Vera

*Aloe vera* is a perennial juicy plant belonging to succulents, commonly used worldwide as a folk remedy to treat different illnesses, including skin disorders. Gel acquired from this plant is widely used in cosmetics, pharmaceuticals, and dietary supplements. Extract of *Aloe vera* contains many potentially pro-health active compounds like anthraquinones, polysaccharides, vitamins, salicylic acid, and plenty of antioxidants, including carotenoids and flavonoids. Some of those elements can attenuate the course of psoriasis by reducing skin itching and alleviating inflammation. In vitro research shows that *Aloe vera* extract (from gel and leaf) can intervene with various proinflammatory pathways, such as inhibiting NF-κB, MAPK, and PI3K signaling and reducing iNOS, IL-6, and IL-1β production macrophages, or decreasing levels of prostaglandin E2 via COX blockade. The research was conducted in a psoriatic model HaCaT cells stimulated TNF-α. The research used doses of 20, 40, and 80 µg/mL of *Aloe Vera* for 24 h, to check cell viability HaCaT cells stimulated with 10 ng/mL TNF-α [85,86].

#### 5.1.2. Artemisia Capillaris

*Artemisia capillaris* has historically been used to treat pyrexia and liver disorders in East Asia as a herbal remedy. This species contains chlorogenic acids, coumarins, and active flavonoid substances exhibiting potential for cancer, hepatitis, malaria, obesity, and pathogenic infection therapies [87]. In the case of psoriasis, extract from *Artemisa capillaris* can potentially inhibit the extensive proliferation of keratinocytes and induce their apoptosis. Furthermore, usage of this plant can presumably reduce leukocyte influx by decreasing expression of ICAM-1 and diminishing nitric oxide levels via inhibiting the production of iNOS [88,89]. The research used doses of 1, 2.5, 5, 10, 25, 50, and 100 μg/mL of *Artemisia capillaris* for 72 h to check the cell viability of HaCaT cells. In further experiments, the scientists only used the dose of 50 μg/mL. Scientists conducted in vitro and in vivo tests on HaCaT cells and mice, which were IMQ stimulated to create a psoriatic model.

#### 5.1.3. St. John’s Wort

St. John’s wort (*Hypericum perforatum*) is traditionally used to treat plenty of conditions, including burns, diarrhea, wounds, sunburn, ulcers, keloid scars, and hemorrhoids [90]. Clinical trials [91] of twenty patients showed that after topical application of the St. John’s wort ointment, acquired scores of redness and the thickness of skin flakes were significantly lower than in the placebo group. The clinical effects of topical *Hypericum perforatum* in plaque-type psoriasis were studied by Mansouri et al. [92]. According to the findings, all variables, including erythema, scaling, and thickness, were substantially reduced in places where the formulated ointment was applied.

#### 5.1.4. Rehmannia Glutinosa

Studies report that extracts from *Rehmannia glutinosa* present potent antioxidative effects by efficiently scavenging free radicals and inhibiting iNOS expression. Moreover, this herb seems to reduce the production of proinflammatory cytokines such as TNF-α, IL-6, IL-17A, and IL-23, decrease PGE2 production via COX2 blockade and suppress the expression of chemoattractants like CCL2 and CXCL10 probably via inhibition of JAK-STAT signaling. In in vivo studies, the researchers used *Rehmannia glutinosa* extract in a dose of 100 and 200 mg/kg b.wt., accordingly, for 7 days, and in in vitro studies, used doses of 0.1, 0.5, and 1.0 mg/mL for 24 h. These results are supported by in vivo studies on mice, where IMQ was used to induce psoriasis-like skin inflammation, and in vitro studies on THP-1 and RAW264.7 cells, where LPS was used to induce psoriasis-like skin inflammation [93,94,95].

#### 5.1.5. Salvia Miltiorrhiza

In terms of anti-inflammatory, antioxidant, antiproliferative properties and their defensive effects, *Salvia miltiorrhiza* and its numerous active constituents have earned significant research attention [95]. However, *Salvia miltiorrhiza* may also have some antipsoriatic capabilities. Previous studies in vitro on HaCaT cells stimulated IL-1, IL-17, IL-22, and oncostatin M and in vivo studies on mouse stimulated IMQ showed that extracts from the root of *Salvia miltiorrhiza* could reduce inflammation by scavenging free radicals and inhibiting Akt and ERK1/2 phosphorylation. In in vitro studies, the researchers used doses of 0.125, 0.25, and 0.5 mmol/L of *Salvia miltiorrhiza*. Furthermore, this herb can thin skin lesions, reduce scales, and inhibit keratinocyte proliferation, simultaneously promoting their apoptosis, which can be important in the treatment of psoriasis. The exact mechanism of action is still to be discovered, nonetheless, it may be connected with inhibition of yes-associated protein (YAP) or/and STAT3 activation [96,97].

### 5.2. Alkaloid

#### Capsaicin

Capsaicin is an active component of chili peppers, and due to its affinity to vanilloid receptors, it can cause depletion of P substance from cutaneous sensory neurons, reducing local vasodilation, angiogenesis, and excessive proliferation of keratinocytes. Additionally, capsaicin can also inhibit NF-κB and AP-1 signaling, influencing inflammation [98] and reducing redness and itching in patients with psoriasis. Nonetheless, topical substance usage is limited due to the pain accompanying its activity. These results are supported by in vitro tests on human promyelocytic leukemia (HL-60) cells stimulated with TPA [99].

### 5.3. Polyphenol

#### 5.3.1. Resveratrol

Resveratrol is a stylben polyphenol derived from grapes, berries, and Polygonum cuspidatum, with known potent anti-inflammatory, anticancer, antidiabetic, and antioxidant effects [100]. In an animal model of imiquimod-induced psoriasis, resveratrol downregulated the production of proinflammatory cytokines such as IL-17-A, IL-19, and IL-23. It promoted keratinocyte death, probably through a mechanism related to activation of SIRT1 and inhibition of Akt kinase. In vitro studies on NHEK cells shows that the proliferation of epidermal keratinocytes can be decreased by using resveratrol. This substance is also responsible for inhibiting aquaporin 3 (AQP3) [101,102].

#### 5.3.2. Curcumin

Curcumin is a component of turmeric and has been used for centuries as a remedy in Southeast Asia. It has antioxidant, anti-inflammatory, antimicrobial, and anticancer effects [103]. Curcumin has been proven as a medicinal substance and even a portion of up to 8 g per day is safe and non-toxic to humans [104]. There is some evidence to support that curcumin can potentially fight psoriasis. Curcumin was discovered to dock at the receptor-binding sites of TNF—by molecular docking in one study. TNF-α needed several residues, including Leu89, Asn90, Asp105, Asn106, and Cys129, to bind to curcumin. This discovery revealed that curcumin interacts directly with TNF-α through non-covalent and covalent interactions. Curcumin can influence or even disrupt signal transduction between TNF-α and its receptor through direct binding, thereby suppressing inflammation induced by this cytokine [105]. Oral curcumin was found to be well tolerated by psoriasis patients in clinical trials. Kurd et al. could not rule out the possibility that the responses were due to a placebo effect or a natural disease reversal because the overall response rate was poor. Nonetheless, two patients showed excellent results, suggesting that massive, placebo-controlled trials would be needed to definitively prove or disprove oral curcumin as a possible psoriasis remedy [106]. Tu et al. [107] conducted in vivo studies and presented on mice that curcumin inhibits TLR2, TLR4, and TLR-9 expression, which may account for a reduction of increased proinflammatory cytokine levels and argument anti-inflammatory IL-10. As a result, their results indicate that curcumin may be used to treat inflammatory diseases as an anti-inflammatory and immunomodulatory agent. Cho et al. [108] using in vitro research, revealed that curcumin inhibits the expression of TNF-induced IL-1ß, IL-6, and TNF in TNF-treated HaCaT cells, as well as TNF-induced cyclin E expression and that the inhibitory effect of curcumin on the expression of these cytokines is likely to be associated with suppression of MAPKs and NF-B, implying that curcumin may be a promising immunomodulatory agent. Curcumin inhibited TNF-induced NF-B activation and IL-6/8 development in HaCaT cells, according to Sun et al. [109]. Curcumin is believed to minimize keratinocyte-related inflammation by inhibiting NF-B activation. Scientists claim there is every reason to believe curcumin can be used to treat psoriasis. The last evidence of curcumin’s effectiveness in psoriasis is that curcumin is a potent inhibitor of phosphorylase kinase activity (PhK); this growth has been correlated with psoriatic activity. PhK levels were analyzed in patients with untreated active psoriasis, in people with receding psoriasis undergoing local treatment, and in healthy subjects. PhK levels in human skin samples showed a direct link with psoriasis activity. In this study, reduced PhK levels were observed in platelet samples treated with a water-alcohol extract from turmeric rhizome. These preliminary observations may suggest that substances capable of inhibiting PhK activity, such as curcumin, could be considered as suitable candidates for local psoriasis treatment [110]. The use of curcumin in psoriasis treatment resulted in the inhibition of keratinocyte proliferation and reduced value of proinflammatory cytokines. This has been tested on in vitro studies on different cells; such a result indicates that turmeric can be used to treat hyperproliferative diseases such as psoriasis [111]. Stimulating cells of the TNF-α HaCaT line and then incubating with curcumin can inhibit TNF’s anti-apoptotic effect, that is, stopping the further development of psoriasis.

#### 5.3.3. Rottlerin

Rottlerin is a natural polyphenolic compound that is purified from *Mallotus phillippinensis.* This compound is reported to exert antihypertensive, antifertility, and antiallergic actions [112]. In vitro studies on HaCaT cells shows that rottlerin is a potent suppressor of keratinocyte proliferation by preventing basal and hydrogen-peroxide-stimulated NF-κB elevation [113]. Min et al. [114] used doses of 0, 1, 5, and 10 μM, and presented that rottlerin not only inhibits keratinocyte (HaCaT and NHEKs cells) proliferation and induces their apoptosis but also significantly blocks secretion of psoriasis driving cytokines including TNF-α, IL-6, IL-17, IL-22, and IL-23.

### 5.4. Flavonoids

#### 5.4.1. Quercetin

Quercetin is a flavonoid that can be found in plants Ginkgo biloba [115] or *Hypericum perforatum* [116]. This flavonoid is plentiful in biological properties, including anti-inflammatory, antioxidant, cardioprotective, vasodilatory, live-protective, and anticancer activities [117]. Kiekow et al. [118] presented that quercetin has anti-inflammatory activity, which is characterized by multiple distinct signaling pathways, including MAPK signaling and NF-κB pathway modulation, discovered through in vitro studies on the C6 cell line-rat glioma cells. Chen et al. [119] used quercetin in doses 30, 60, and 120 mg/kg for 7 days to investigate its influence on cytokine levels. They demonstrated via in vivo studies of psoriatic models and IMQ-stimulated mice that the levels of TNF-α, IL-6, and IL-17 were significantly decreased after administration of different doses of quercetin.

#### 5.4.2. Apigenin

Apigenin is a natural flavonoid. It is present in a large variety of food products, including sweet pepper, parsley, thyme, celery, onions, and tea [120]. This flavonoid has antibacterial, anti-inflammatory, and antioxidant properties. Apigenin, a non-mutagenic plant flavone, is a strong inhibitor of NF-κB activation in autoimmune cells [121]. Miroeva et al. [122] used 5 μmol of apigenin using in vivo studies and showed that IL-6 and IL-12 levels decreased after apigenin stimulation in mice. The levels of these cytokines are high in psoriasis.

#### 5.4.3. Kaempferol

Kaempferol is a flavonoid that is abundantly found in fruits and vegetables; mainly, we can find it in tea, broccoli, apples, strawberries, and beans [123]. Polyphenols, such as kaempferol, have important effects on inflammation-induced diseases like psoriasis pathogenesis due to their anti-inflammatory properties. Liu et al. [124] demonstrated that in the psoriatic skin lesion, kaempferol undermined the psoriatic skin lesion and inflammation and decreased gene expression of primary proinflammatory cytokines. In in vivo studies, the researchers induced a psoriasis model in mice with used IMQ. The mice were orally administered 50 and 100 mg/kg of kaempferol for 7 consecutive days. They observed that kaempferol inhibited Th17 levels and stifled the phosphorylation of NF-κB, which is one of many typical proinflammatory signals in psoriasis.

#### 5.4.4. Genistein

Genistein is a flavonoid ordinarily found in various vegetables, such as soybeans and fava beans. Its concentration in food amounts from 1 to 2 mg/g [125]. A broad spectrum of biological effects such as antioxidant, antiangiogenic, and anticancer activity has been documented in several studies [126]. Wang et al. [127] noticed in vivo studies that genistein, in doses 50 and 100 μM for 2 h in a psoriasis-model in mice, causes a reduction of cytokine expressions such as IL-1β, IL-6, TNF-α, CCL2, IL-17, and IL-23. They also discovered that in IMQ-treated mouse skin and TNF-alpha-stimulated HaCaT cells, genistein suppressed STAT3 phosphorylation and also inhibited Iβ-phosphorylation and nuclear NF-alpha-translocation in TNF-alpha-stimulated HaCaT cells. Smolińska et al. [128], through in vitro studies, discovered that genistein in a dose of 1 μg/mL for 24 h of incubation stifled the ROS generation in HaCaT cells, which were by stimulated TNF-α or LPS. Studies indicate that genistein in the psoriatic model can attenuate ROS-mediated NF-κB activation and NF-κB-dependent inflammatory cytokine production (keratinocytes stimulated TNF or LPS).

#### 5.4.5. Rutin

Rutin is a polyphenolic hydrophobic compound that belongs to the flavonoid family present in various foods, such as citrus, apples, Betula leaves, buckwheat, black tea, and green tea [129]. This substance is implicated in antioxidant and anti-inflammatory processes and has been used in several experiments aimed at evaluating its potential as an active ingredient in medicinal products [130]. Rutin is found in *Memecylon malabaricum*, a small tree with blue flowers. Researchers investigated the antipsoriatic activity of rutin at a dose of 100 mg/mL in vivo studies in a mouse tail test and in vitro antipsoriatic activity using HaCaT cells. They discovered that the plant, which showed strong activity in the mouse tail test for psoriasis, did not show good activity in any of the three in-vitro tests they conducted. *Memecylon malabaricum* demonstrated modest activity in the LOX inhibition assay, in addition to strong in vivo activity. Their results demonstrate that the whole *M. malabaricum* leaf has the antipsoriatic ability, confirming its traditional use by Siddha healers [131].

#### 5.4.6. Naringenin

Naringenin is one of the flavanones found in fruits such as grapefruit, lemon, tangerine, and orange [132]. This substance is abundant in many pharmacological activities such as anticancer, antioxidant, anti-inflammatory, antibacterial, and cardioprotective activities [133]. Martinez et al. [134] observed in in vivo studies that naringenin, compared with light (UVB), significantly reduces the levels of cytokines TNF-α, IL-1β, and IL-6, which causes the inflammatory effect in the psoriasis model.

#### 5.4.7. Naringin

Naringin usually occurs in citrus plants, such as grapefruit, orange or cooked tomato paste, cherries, beans, and oregano [135,136]. This flavonoid has numerous therapeutic benefits associated with psoriasis pathogenesis, such as anti-inflammatory activity and inhibitory activity of chemokine production [137]. Deenonpoe et al. [138] discovered in clinical trials on 20 patients that naringin exposed an inhibitory effect on TNF-α and IL-6 production, and these cytokines at a high-level cause immunological effects in psoriasis.

#### 5.4.8. Epigallocatechin-3-gallate (EGCG)

EGCG belongs to the flavonoids. Its high concentration is found in green tea, quercetin, theaflavin, and thearubigin, present in black tea, as well as tannic acid [139]. It has been reported that EGCG possesses many benefits. This flavonoid has anti-inflammatory, antitumor, antioxidant, and anti-ultraviolet radiation effects. Zhang et al. [140] conducted in vivo research in which they used doses of 0.25 g and 12.5 mg for 6 days on mice that were IMQ-stimulated to create the psoriasis model. They found that local application of EGCG can decrease PCNA (proliferating cell nuclear antigen) expression, successfully inhibit IMQ-induced abnormal proliferation of epidermal cells, and alleviate psoriasis signs. They also showed that the activity of SOD and CAT decrease after using EGCG. The results demonstrated that EGCG could improve the symptoms of mice psoriasis by regulating antioxidant factors.

#### 5.4.9. Anthocyanidins

Anthocyanins are green water-soluble flavonoids. The compounds accountable for the colors red, purple, and blue, are found in fruit and vegetables. We can find them in berries, strawberries, currants, grapes, and other tropical fruits. Likewise, aubergine skin and red cabbage are abundant in a high amount of anthocyanins [141]. This flavonoid has many benefits; it has anti-inflammatory, radiation protection, and antioxidant effects [142]. Chamcheu et al. [143] conducted in vivo research and found that delphinidin (anthocyanins) therapy suppressed the expression of proliferation and inflammation while simultaneously inducing epidermal differentiation markers in a psoriatic model. They also observed that delphinidin inhibits the increased release of psoriatic-associated inflammatory cytokines.

### 5.5. Carotenoid

#### Lycopene

The lycopene is a carotenoid the most abundant in tomatoes. Other sources of lycopene include red fruits such as rosehips, watermelons, red grapefruits, papayas, apricots, and pink guavas, among others [144,145]. In vitro and in vivo studies have suggested promising anti-inflammatory, antiangiogenic, anti-invasive, and antimetastatic effects of lycopene [146]. In in vitro and in vivo (in mice) studies, Shih et al. [147] found that lycopene inhibited IMQ-induced psoriasis-like inflammation in keratinocytes. The researchers conducted the topical treatment with a dose of 0.12 mg/mL lycopene for 7 days and the oral treatment with a dose of 0.12 mg/kg for 42 days. Furthermore, local lycopene treatment reduced monocytic cell adhesion in an IMQ-induced psoriasis-like dermatitis mouse model, which not only offered local symptomatic relief but also led to decreased monocytic cell adhesion.

### 5.6. Anthraquinone

#### Emodin

Emodin is an anthraquinone derivative isolated from Chinese herbs, including *Rheum palmatum*, *Polygonum cuspidatum*, *Polygonum multiflorum*, *Aloe vera*, and *Cassia obtusifolia* [148]. It possesses a wide range of pharmacological activities, including anticancer, hepatoprotective, anti-inflammatory, antioxidant, and antibacterial effects. In vitro studies confirmed that topical application of a natural compound combination of herbs at a dose 10 μM, which included emodin, lowered the proliferation rate of IL-22-stimulated keratinocytes and relieved IMQ-induced psoriasis-like dermatitis [149].

## 6. Conclusions

Hitherto, patients with psoriasis have not had access to a remedy that cures that condition. Some preparations inhibit the action of immune factors or suppress the effects of psoriasis. Current therapeutic substances possess certain drawbacks, including the frustration of the patients due to the ineffectiveness of drugs and possible side effects—mood swings, diarrhea, and vomiting. There is a lack of an effective and long-term treatment plan in the fight against psoriasis. There is a great need for the continuous development of new, safe, and effective treatment of psoriasis. Among the many active compounds that have been studied for the relief of psoriasis, extracts from plants and specific phytochemicals from natural resources have been of great interest in recent decades. Several studies evaluating psoriasis therapy based on natural sources revealed potential activity, especially antiproliferative effects, reduction of itching, and lowering the levels of inflammation cytokines. Natural substances, in comparison with medicament, do not cause frustration of the patients, mood swings, diarrhea, and vomiting, which is the positive side of their use. To date, most reports regarding the antiproliferative efficacy of natural compounds in the treatment of psoriasis are based on laboratory work or animals. Some studies suggest the use of natural products in psoriasis treatment, only because of their ability to inhibit the proliferation of keratinocytes. Evidence from in vitro cellular studies, in vivo animal testing, and clinical trials offer a lot of information on natural products’ success in treating psoriasis (Table 1). All the studies discussed in this review reveal the benefits of the used substances without side effects. Furthermore, researchers observed the enhancement in immune system response, as well as reduction of oxidative stress after the treatment. Further in vitro, in vivo, and clinical trials can confirm results that natural substances could be a potential and helpful candidate that improves the clinical outcome of patients treated with systemic treatments, either classical or immunobiological.

## Figures and Tables

**Table 1:** The table presents different chosen plants and phytochemicals with their application.

Phytochemicals	Classification	Natural Occurrence	Application with References
*Aloe vera*	Vegetal medicinal species	-	[85,86]
*Artemisia capillaris*	Vegetal medicinal species	-	[88,89]
St. John’s wort *Hypericum perforatum*	Vegetal medicinal species	-	[92]
*Rehmannia glutinosa*	Vegetal medicinal species	-	[93,94,95]
*Salvia miltiorrhiza*	Vegetal medicinal species	-	[96,97]
Capsaicin	Alkaloid	chili peppers	[98,99]
Resveratrol	Polyphenol	red grapes, peanuts, berries	[101,102]
Curcumin	Polyphenol	-	[105,106,107,108,109,110,111]
Rottlerin	Polyphenol	-	[113,114]
Quercetin	Polyphenol, flavonoids	onion, apple, broccoli, citrus fruits, cherries, green tea, coffee, red wine, capers	[118,119]
Apigenin	Polyphenol, flavonoids	sweet pepper, parsley, thyme, celery, onions, tea	[121,122]
Kaempferol	Polyphenol, flavonoids	tea, broccoli, apples, strawberries, beans	[124]
Genistein	Polyphenol, flavonoids	soy beans, fava beans	[127,128]
Rutin	Polyphenol, flavonoids	citrus, apples, Betula leaves, buckwheat, black tea, green tea	[131]
Naringenin	Polyphenol, flavonoids	grapefruit, lemon, tangerine, orange	[134]
Naringin	Polyphenol, flavonoids	citrus, cooked tomato paste, cherries, beans, and oregano	[138]
Epigallocatechin-3-gallate (EGCG)	Polyphenol, flavonoids	green tea, black tea	[140]
Anthocyanin	Polyphenol, flavonoids	berries, strawberries, currants, grapes, tropical fruits, aubergine skin, red cabbage	[143]
Lycopene	Carotenoid	tomatoes, rosehips, watermelons, red grapefruits, papayas, apricots, and pink guavas	[147]
Emodin	Anthraquinone	rhubarb, water pepper	[149]

## Data Availability

Not applicable.
